# Metabolic syndrome and coronary artery bypass graft surgery

**DOI:** 10.1186/cc9425

**Published:** 2011-03-11

**Authors:** M Brouard, JJ Jimenez, JL Iribarren, N Perez, L Lorente, P Machado, JM Raya, R Perez, JM Borreguero, R Martinez, ML Mora

**Affiliations:** 1 Hospital Universitario de Canarias, La Laguna, Spain

## Introduction

Metabolic syndrome (MS) is a constellation of disorders that increases the risk for coronary heart disease. This study was conducted to examine the incidence of metabolic syndrome in coronary artery bypass graft (CABG) patients and to determine if metabolic syndrome affects clinical outcomes in the perioperative setting.

## Methods

A cohort study of elective CABG surgery patients. Metabolic syndrome was defined using recent established criteria [1]. Demographic variables, comorbid conditions, surgical procedures and postoperative variables were collected. SPSS 15 was used.

## Results

We studied 508 patients. MS was defined in 333 (66%) patients, 241 (72%) males and 92 (28%) females, mean age 66 ± 9 years. MS had greater glucose levels at all postoperative time points (*F*: 41.6, *P *< 0.001), higher leptins levels (*F*: 4.7, *P *< 0.044), higher thrombomodulin at 0 hours and 4 hours after surgery (*F*: 6, *P *= 0.016), and lower 24-hour-postoperative blood loss after adjusting by tranexamic acid (*F*: 4.6, *P *= 0.032). MS had higher incidence of renal dysfunction (RIFLE: I) 13 (4%) versus 1 (0.6%) (*P *= 0.027).

## Conclusions

MS was associated with a procoagulant state that may decrease postoperative blood loss. Nevertheless MS was associated with worse adverse events as renal dysfunction.
